# Identifying the pulsed neuron networks’ structures by a nonlinear Granger causality method

**DOI:** 10.1186/s12868-020-0555-z

**Published:** 2020-02-12

**Authors:** Mei-jia Zhu, Chao-yi Dong, Xiao-yan Chen, Jing-wen Ren, Xiao-yi Zhao

**Affiliations:** 1 School of Electric Power, Inner Mongolia University of Technology, Hohhot, 010080 China; 2 Inner Mongolia Key Laboratory of Mechanical and Electrical Control, Hohhot, 010051 China

**Keywords:** Integrate-and-fire model, Radial basis function, Nonlinear granger causality, Network structure identification

## Abstract

**Background:**

It is a crucial task of brain science researches to explore functional connective maps of Biological Neural Networks (BNN). The maps help to deeply study the dominant relationship between the structures of the BNNs and their network functions.

**Results:**

In this study, the ideas of linear Granger causality modeling and causality identification are extended to those of nonlinear Granger causality modeling and network structure identification. We employed Radial Basis Functions to fit the nonlinear multivariate dynamical responses of BNNs with neuronal pulse firing. By introducing the contributions from presynaptic neurons and detecting whether the predictions for postsynaptic neurons’ pulse firing signals are improved or not, we can reveal the information flows distribution of BNNs. Thus, the functional connections from presynaptic neurons can be identified from the obtained network information flows. To verify the effectiveness of the proposed method, the Nonlinear Granger Causality Identification Method (NGCIM) is applied to the network structure discovery processes of Spiking Neural Networks (SNN). SNN is a simulation model based on an Integrate-and-Fire mechanism. By network simulations, the multi-channel neuronal pulse sequence data of the SNNs can be used to reversely identify the synaptic connections and strengths of the SNNs.

**Conclusions:**

The identification results show: for 2–6 nodes small-scale neural networks, 20 nodes medium-scale neural networks, and 100 nodes large-scale neural networks, the identification accuracy of NGCIM with the Gaussian kernel function was 100%, 99.64%, 98.64%, 98.37%, 98.31%, 84.87% and 80.56%, respectively. The identification accuracies were significantly higher than those of a traditional Linear Granger Causality Identification Method with the same network sizes. Thus, with an accumulation of the data obtained by the existing measurement methods, such as Electroencephalography, functional Magnetic Resonance Imaging, and Multi-Electrode Array, the NGCIM can be a promising network modeling method to infer the functional connective maps of BNNs.

## Background

It is well known that large number of neurons interacted with specific and efficient connections compose of complex Biological Neural Networks (BNN) [1], which are controlling and coordinating a series of life activities of the human bodies. The major characteristics of BNNs, for example, Integrate-and-Fire (IF) mechanism, plasticity of synapses, and the complexity of network structure, enable them to have adaptability and learning ability, which are significantly different from general artificial networks. These unique characteristics of the BNNs constitute the internal regulatory mechanism and substantial basis of various life functions. Therefore, it is of great significance to explore the connection mode and connection characteristics of BNNs for studying the information processing and transmission mechanism of BNNs. At present, this research objective is still restricted by two factors: (1) Accurate identification of network structure requires a large amount of multi-channel neuronal pulse response data with a high temporal and spatial resolution. However, the data quality obtained by the existing measurement methods, such as Electroencephalography (EEG), Magnetoencephalography (MEG), functional Near-infrared Spectroscopy (fNIRS), functional Magnetic Resonance Imaging (fMRI), and Invasive Electrode Implantation (IEI), are usually limited because of a low temporal and spatial resolution. (2) Because biological neurons have strong nonlinear dynamic characteristics, currently, there are few effective network structure reverse identification methods, which can accurately model and adapt this nonlinear dynamic relationship.

In recent years, Multi-Electrode Array (MEA) technology has developed rapidly [2] and gradually become an efficient method that can simultaneously measure the electrical activity of multiple neurons in in-vitro cultured BNNs. The data obtain by MEAs has a high temporal resolution and spatial resolution, compared to the afore-mentioned invasive or noninvasive measurement methods. The MEAs allow synchronous recording the electrical activities of multiple neurons in million second level, and the relationship between neuron activities in different channels is obtained through correlation analysis of the potential sequences of each channel. The development of this new technologies greatly promotes the research on the identification of the functional connection structures of BNNs. Many researchers apply linear dynamics, informatics, probability statistics and other theories to propose various algorithms to identify the structures of BNN, such as Mutual Information (MI) [3], Direction Transfer Function (DTF) [4], Dynamic Bayesian Network (DBN) [5], Evolutionary Mapping Approach (EMA) [6], Dynamical Causal Modeling (DCM) [7]. Although these methods can solve the identification problem of network information flow to a certain extent, they still have some limitations in practical applications. For example, DTF is a hypothesis testing process based on parameters in a linear Auto-regression (AR) model, which is not suitable for data processing of essentially nonlinear networks. EMA under an assumption of weak coupling between different channels, extract the phase information of the data to discern the coupling strength and the directions between two channel data. Therefore, EMA is difficult to be extended to multichannel analysis. DBN can be used to process short-term bioinformatics data with noise, however, its application to the identification of BNNs is rarely reported. In contrast with the previous methods, the DCM methods have two remarkable advantages: they extract more useful network connective information only from the available multi-channel data by computing two correlation matrices; they effectively resist noise contamination with unknown statistics of noises. However, the DCMs are mainly based on linearized ODE models, which usually require the dynamical functions are differentiable at steady states. That is not the case for the Integrate and Fire dynamics of BNNs, which are commonly considered as nondifferentiable and nonlinear [7, 8]. In this article, a Nonlinear Granger Causality Identification Method (NGCIM) is used to identify the structure of BNN with multiple neurons [9]. Considering a significant nonlinear dynamical property of biological neurons, we use a Radial Basis Functions (RBF) to fit neuron’s IF dynamics of Spiking Neural Networks (SNN). Thus, the functional connections can be identified by investigating the nonlinear Granger causality between the neurons in the SNNs.

## Results

To verify the effectiveness of the proposed method in multi-channel BNN analysis, the NGCIM based on the RBF is applied to the network structure identifications. The SNNs can simulate the dynamic process of biological neurons’ discharge to mimic the dynamic behavior and physiological mechanism of BNNs in a certain accuracy [10]. The IF model of one neuron can be expressed by the following first-order differential equation:

1 $$I\left(t\right)=\frac{V\left(t\right)-{E}_{m}}{{R}_{m}}+{C}_{m}\frac{dV}{dt}$$

It can be transformed to:

2 $${\tau }_{m}\frac{dV}{dt}={E}_{m}-V\left(t\right)+{R}_{m}I(t)$$

where ${\tau }_{m}={R}_{m}{C}_{m}$ is the time constant for the establishing process of membrane voltage, ${C}_{m}$ is the membrane capacitance, ${R}_{m}$ is the membrane resistance, ${E}_{m}$ is the resting potential, and *I*(*t*) is the sum of the synaptic currents generated by the firing pulses of the pre-synaptic neurons. The sum of the synaptic currents can be expressed as:

3 $$I(t)=\sum_{j}{\omega }_{ij}\sum_{f}\alpha (t-{t}_{ j}^{(f )})$$

where $\alpha (t-{t}_{ j}^{(f )})$ represents the effect function of presynaptic neurons’ firing on postsynaptic neurons, in a form of negative exponential decay. The notation ${t}_{ j}^{(f )}$ represents the moment when a presynaptic neuron *j* emits its behavioral potential. The multi-channel neuronal firing sequence, generated by SNN network simulations [11], is used to reversely identify the causal synaptic connections and action strength existing in the network [12, 13].

**Fig. 1:**
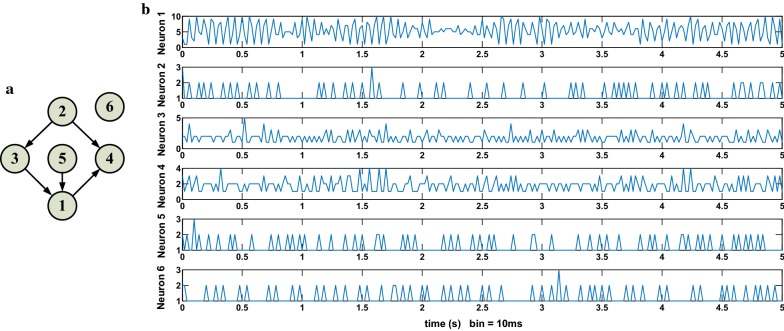
A 6-mode SNN simulation (**a**) 6-node SNN’s structure (**b**) multivariate response data generated by the network simulation (after sampling the pulse sequences of the neurons)

**Fig. 2:**
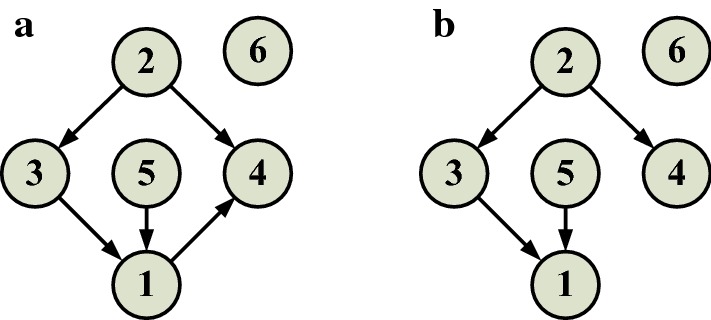
Identification results of 6 node network structure by the LGCIM and the NGCIM. **a** The network connection structure identified by the NGCIM with the Gaussian kernel function. **b** The network connection structure identified by the LGCIM

A single biological neuron is regarded as a node, and multiple interactions between biological neurons, such as electrical and chemical signal transmission, are represented by the directed edges with arrows. To simulate the real BNNs, where synaptic connections are highly sparse, the connection ratio of the network is set at 0.2, i.e., each neuron is only connected to 20% of other neurons in the networks [14]. Firstly, the network connection matrix B is generated randomly, where "1" means there is a direct connection between the two nodes, and "0" means there is no direct connection between the two nodes. The interaction between neurons in the networks is determined according to the principle of "column acts row". As shown in Fig. 1a, there are five direct connections among the six nodes of the pulse neuron network. The pulse sequences of the neurons were sampled at an interval of 10 ms (only the first 5 s were shown). See Fig. 1b for the multivariate response data. The proposed NGCIM is applied to detect 30 conditional nonlinear Granger causality between 6 neurons listed in Table 1, where the notation “→” represents the direct effect of presynaptic neurons on postsynaptic neurons, and the notation $"/"$ indicates “under the condition of the neurons of”. For example, “1$\to 2$/3, 4, 5, 6” represents that under the condition of the set of neuron 3, 4, 5, and 6, neuron 1 has an effect on neuron 2. A Linear Granger Causality Identification Method (LGCIM) and a NGCIM with a Gaussian kernel function are respectively used to detect the conditional Granger causality of the 30 directed connections. The identification results are shown in Fig. 2.

**Table 1:** The conditional nonlinear Granger causality in the 6 neuron network

Notation relation	Notation relation	Notation relation	Notation relation
1 (1 $\to 2$/3,4,5,6)	2 (1 $\to 3$/2,4,5,6)	3 (1 $\to 4$/2,3,5,6)	4 (1 $\to 5$/2,3,4,6)
5 (1 $\to 6$/2,3,4,5)	6 (2 $\to 1$/3,4,5,6)	7 (2 $\to 3$/1,4,5,6)	8 (2 $\to 4$/1,3,5,6)
9 (2 $\to 5$/1,3,4,6)	10 (2 $\to 6$/1,3,4,5)	11 (3 $\to 1$/2,4,5,6)	12 (3 $\to 2$/1,4,5,6)
13 (3 $\to 4$/1,2,5,6)	14 (3 $\to 5$/1,2,4,6)	15 (3 $\to 6$/1,2,4,5)	16 (4 $\to 1$/2,3,5,6)
17 (4 $\to 2$/1,3,5,6)	18 (4 $\to 3$/1,2,5,6)	19 (4 $\to 5$/1,2,3,6)	20 (4 $\to 6$/1,2,3,5)
21 (5 $\to 1$/2,3,4,6)	22 (5 $\to 2$/1,3,4,6)	23 (5 $\to 3$/1,2,4,6)	24 (5 $\to 4$/1,2,3,6)
25 (5 $\to 6$/1,2,3,4)	26 (6 $\to 1$/2,3,4,5)	27 (6 $\to 2$/1,3,4,5)	28 (6 $\to 3$/1,2,4,5)
29 (6 $\to 4$/1,2,3,5)	30 (6 $\to 5$/1,2,3,4)

It can be found that the NGCIM can identify all the interactions between all nodes correctly, however, LGCIM fails to identify the connection from node 1 to node 4. Thus, the accuracy of identification of LGCIM is 97.22%. To more effectively validate the two methods, ten rounds of simulation and identification are carried out for small-scale networks with 2–6 nodes. In each round, 100 randomly connected network are established with a sparse connective ratio. Finally, the average identification accuracies of the 10-round simulations are shown in Table 2. The network structure identifications are further extended to the SNNs’ structures of 20 and 100 nodes, and the identification accuracies of 10 rounds of identifications are shown in Tables 3 and 4 respectively.

**Table 2:** The average identification accuracy of 10-round simulations of small-scale networks with 2–6 nodes

Number of nodes	2 nodes (%)	3 nodes (%)	4 nodes (%)	5 nodes (%)	6 nodes (%)
LGCIM	100	99.53	98.03	97.60	97.26
NGCIM	100	99.64	98.64	98.37	98.31

**Table 3:** The average identification accuracies of the 20-nodes SNNs

Rounds	1 (%)	2 (%)	3 (%)	4 (%)	5 (%)	6 (%)	7 (%)	8 (%)	9 (%)	10 (%)
LGCIM	82.88	82.05	82.58	82.90	82.39	82.64	82.49	82.04	82.53	82.92
NGCIM	84.84	84.42	85.03	84.64	84.89	85.36	84.99	84.77	84.82	84.95

**Table 4:** The average identification accuracies of 100-node SNNs

Rounds	1 (%)	2 (%)	3 (%)	4 (%)	5 (%)	6 (%)	7 (%)	8 (%)	9 (%)	10 (%)
LGCIM	80.20	80.23	80.18	80.19	80.26	80.18	80.17	80.21	80.22	80.25
NGCIM	80.54	80.45	80.75	80.23	80.44	80.73	80.71	80.75	80.32	80.66

It can be summarized from Tables 2, 3, 4: 1. As the scale of the SNNs increases, the identification accuracies of both linear and nonlinear methods decrease, but the declining trend gradually becomes stable and goes into a plateau. 2. The identification accuracies of NGCIM based on the Gaussian kernel function for the small-scale networks with 2–6 nodes are achieved respectively 100%, 99.64%, 98.64%, 98.37%, and 98.31%, which are significantly higher than those of LGCIM, which are 100%, 99.53%, 98.03%, 97.60%, and 97.26%. For the medium-scale networks with 20 nodes and the large-scale networks with 100 nodes, the identification accuracies of the NGCIM is 84.87% and 80.56%, which are still significantly higher than 82.54% and 80.21% of the LGCIM for the same scale networks. The fact reflects that the accuracies of the NGCIM based on the Gaussian function is significantly higher than those of the LGCIM during identifying SNNs’ connected structures. In addition, the NGCIM is also used to identify SNNs’ connection structures when the RBF select the different kernel functions: Gauss Function (GF), Reflected Sigmoidal Function (RSF), IMQF (Inverse Multi-quadrics Function). The identification results with different kernel functions is shown in Table 5.

**Table 5:** The average accuracies of 10-round identifications with three different kernel functions

	2 nodes (%)	3 nodes (%)	4 nodes (%)	5 nodes (%)	6 nodes (%)	20 nodes (%)	100 nodes (%)
GF	100	99.64	98.64	98.37	98.37	84.87	80.56
RSF	100	99.63	98.34	98.23	98.31	84.85	80.49
IMQF	100	99.67	98.76	98.53	98.47	85.11	80.88

Because a large amount of computation costs, especially for 100-node networks, the NGCIM is coded and assigned to an *AMAX* GPU server with a *Nvidia Tesla* K40 card. The time consumed by the NGCIM with three different kernel functions are shown in the Table 6, for the 10 rounds of the different scales of network simulations.

**Table 6:** The time consumed by 10-round identifications with three different kernel functions

	2 nodes (s)	3 nodes (s)	4 nodes (s)	5 nodes (s)	6 nodes (s)	20 nodes (s)	100 nodes (s)
GF	298	472	625	792	972	3597	49,234
RSF	300	483	657	793	994	3615	53,647
IMQF	308	494	640	806	1015	3633	54,266

In each round of simulations, 100 randomly connected networks are established and identified

It can be found that IMQF ranks as the highest average accuracies of 10-round identifications by NGCIM, then GF as the second highest and RSF as the lowest. However, for computational speeds, GF is the fastest among the three kernels. IMQF consumes the largest amount of time because of its relatively higher computational complexity.

## Discussion

In the process of identifying the structures of BNNs, the traditional LGCIM has some limitations due to the essential non-linearity of biological neurons. It is necessary to extend the network model to the nonlinear model and establish the conditional nonlinear Granger causality detection method, i.e., NGCIM.

In the NGCIM, the nonlinear dynamic effects between neurons were fitted by the RBFs, which commonly consist of three types of nonlinear kernel functions. For testing the proposed NGCIM, neuron firing behaviors were simulated by artificial SNN model based on the IF mechanism, and both LGCIM and NGCIM are applied to the multi-channel neuronal pulse sequence data generated by network simulations. For the 2–6 nodes (small-scale) SNNs, the 20 nodes (the middle-scale) SNNs, the 100 nodes (large-scale) SNNs, the 10 rounds of 100 randomly connected network structures were formed and simulated. Then, the causal synaptic connections and strength in the network are identified reversely.

## Conclusions

BNN is one of the most complex nonlinear systems ever discovered by human till the present time. Drawing the connection structure maps of brain networks has more crucial theoretical significance for the researches of neurophysiology and pathology, and even helps to create more higher-level artificial intelligent systems.

The NGCIM is applied to the network structure discovery process of the SNN simulation models based on IF mechanism. The multi-channel spike sequence data are generated by the network simulations. The method can use the simulated data to reversely identify the synaptic connections and their strengths existing in the networks. The identification results show that the average identification accuracy of the NGCIM based on RBF is significantly higher than that of the LGCIM, which verifies the effectiveness of the proposed method in the task of BNNs structure identification. The comparisons between three different kernel functions show IMQF has the highest identification accuracy but consume the longest computational time, especially for the 100-node SNNs. Such a relatively heavy burden of computational task can be assigned to the GPU server for parallel distributed computations.

The development of Electroencephalography, functional Magnetic Resonance Imaging, and Multi-Electrode Array greatly promoted the research on the identification of the functional connection structures of BNNs. NGCIM is compatible to the nonlinear essences of BNN spike firings than the other previous methods are. Therefore, with an accumulation of the data obtained by the existing measurement methods, the NGCIM can be a promising network modeling method to infer the functional connective maps of BNNs.

## Methods

### Liner Granger causality

Granger first proposed the concept of causality in 1969 to detect causality relationships between two simultaneously recorded signals [15]. The processes become one of the most attracting scientific investigations in time series analysis. Thereafter, a variety of applications arose in different fields, such as economics, physiology, neuroscience, and many others [16]. If the prediction of one time series can be improved by incorporating measurements from the second time series in a regression model, then the second time series is said to have a “Granger causality” on the first time series.

The nonlinear multivariate Granger causality analysis is originally derived from a definition and test of linear Granger causality in a two-variate system, which is commonly based on a Vector AutoRegressive (VAR) model [17]. Take an example of two stationary time series of *N* simultaneously measured quantities {${x}_{k}$}, *k* = 1, 2, …, *N* and {${y}_{k}$}, *k* = 1, 2, …, *N*. A VAR model can be constructed as:

4 $${x}^{k}-{{\mathbf{V}}}_{11}{{\varvec{X}}}^{ k}-{{\mathbf{V}}}_{12}{{\varvec{Y}}}^{ k}={\varepsilon }_{1}, \quad {\text{var}}\left[{\varepsilon }_{1}\right]={\varSigma }_{1} , \quad \left(k=1, 2, \ldots N-m-\tau \right)$$

5 $${y}^{k}-{{\mathbf{V}}}_{21}{{\varvec{X}}}^{ k}-{{\mathbf{V}}}_{22}{{\varvec{Y}}}^{ k}={\eta }_{1}, \quad  {\text{var}}\left[{\eta }_{1}\right]={H}_{1} , \quad \left(k=1, 2, \ldots N-m-\tau \right)$$

where (${x}^{k}, {y}^{k}, {{\varvec{X}}}^{ k}, {{\varvec{Y}}}^{ k}$) are realizations of the stochastic variates (*x*, *y*, ***X***, ***Y***) and ${x}^{k}= {x}_{k+m+\tau }$, ${y}^{k}= {y}_{k+m+\tau }$, ${{\varvec{X}}}^{ k}=({x}_{k+m-1},{x}_{k+m-2}$,…,${x}_{k}$)^T^, ${{\varvec{Y}}}^{ k}=({y}_{k+m-1},{y}_{k+m-2}$,…,${y}_{{k}}$)^T^. The notation *m* denotes an order of the model and $\tau$ is a step of a pure delay. ${{\mathbf{V}}}_{11},$ ${{\mathbf{V}}}_{12},$ ${{\mathbf{V}}}_{21},$ and ${{\mathbf{V}}}_{22}$ are *m*-dimensional row vectors, which represent the weights of individual components in ${{\varvec{X}}}^{ k}$ and ${{\varvec{Y}}}^{ k}$ contributing to a prediction of ${x}^{k}$ and ${{y}}^{{k}}$. The prediction errors of the two variates are ${\varepsilon }_{1}$ and ${\eta }_{1}$ and their variances can be represented as ${\varSigma }_{1}$ and ${H}_{1}$. For simplicity, a shorthand of the two-variate VAR model in a form of random variates is described as:

6 $$x-{{\mathbf{V}}}_{11}{\varvec{X}}-{{\mathbf{V}}}_{12}{\varvec{Y}}={\varepsilon }_{1}, \quad {\text{var}}[{\varepsilon }_{1}]={\varSigma }_{1}$$

7 $$y-{{\mathbf{V}}}_{21}{\varvec{X}}-{{\mathbf{V}}}_{22}{\varvec{Y}}={\eta }_{1}, \quad {\text{var}}[{\eta }_{1}]={H}_{1}$$

Without any interactions between the two variates, the VAR model is then deduced to:

8 $$x-{{\mathbf{W}}}_{1}{\varvec{X}}={\varepsilon }_{2}, \quad {\text{var}}[{\varepsilon }_{2}]={\varSigma }_{2}$$

9 $$y-{{\mathbf{W}}}_{2}{\varvec{Y}}={\eta }_{2}, \quad {\text{var}}[{\eta }_{2}]={H}_{2}$$

where ${{\mathbf{W}}}_{1}, {{\mathbf{W}}}_{2}$ are *m*-dimensional weight vectors and ${\varepsilon }_{2}$, ${\eta }_{2}$ are the prediction errors of each variate by its past values.

According to the thought of Granger causality, if the prediction of *x* is improved by incorporating the past values of *y*, then *y* has a causal influence on *x*. Thus, a Granger causality of *y* on *x* can be evaluated as:

10 $${\text{F}}_{y\to x}={\text{ln}}\frac{{\varSigma }_{2}}{{\varSigma }_{1}}$$

If *x* and *y* are independent of each other, then ${\mathbf{V}}_{12}$ and ${\mathbf{V}}_{21}$ are both zero vector. Models (6) and (7) become models (8) and (9). Thus, ${\varSigma }_{2}={\varSigma }_{1},{\text{ and F}}_{y\to x}=0$. In another case that *y* has a causal effect on *x*, then ${\varSigma }_{2}>{\varSigma }_{1}$, so that ${\text{F}}_{y\to x}>0$. Similarly, we can define the measure of the Granger causality of *x* on *y*:

11 $${\text{F}}_{x\to y}={\text{ln}}\frac{ {H}_{2}}{{H}_{1}}$$

If ${\text{F}}_{x\to y}=$0, then *x* has no causal effect on *y*. While ${\text{F}}_{x\to y}>$0, *x* has a causal effect on *y*.

### RBFs for nonlinear modeling

Currently, BNN is one of the most complex nonlinear network systems as human knows [18]. In the process of identification of BNN structures, how to conduct a nonlinear network analysis in a framework of linear Granger causality still has a crucial theoretical value and practical significance. RBFs, whose linear combinations can approximate any nonlinear function, are commonly employed to fit the dynamic causal relationship among nonlinear network variates [19]. A RBF is defined as a real valued function of a vector ***X*** that depends on the distance from the origin: $\boldsymbol{\Phi }$(${\varvec{X}}$) = $\boldsymbol{\Phi }$($\Vert {\varvec{X}}\Vert$) or depending on an distance to any center *c*, $\boldsymbol{\Phi }$(${\varvec{X}}-c$) = $\boldsymbol{\Phi }$($\Vert {\varvec{X}}-c\Vert$). The notation *r* = $\Vert {\varvec{X}}- c\Vert$ represents a modulus, or the norm of 2, of the difference vector. Usually, $\boldsymbol{\Phi }$(${{r}}$) can takes the following forms:

1) GF: $$\boldsymbol{\Phi }(r)$$ = exp ($$-{r}^{2}/2{\sigma }^{2}$$).

2) RSF: $$\boldsymbol{\Phi }(r)=1/\left(1+{\text{exp}}({r}^{2}/{\sigma }^{2})\right)$$

3) IMQF: $$\boldsymbol{\Phi }(r)=$$1/$$\sqrt{{r}^{2}+{\sigma }^{2}}$$

**Fig. 3:**
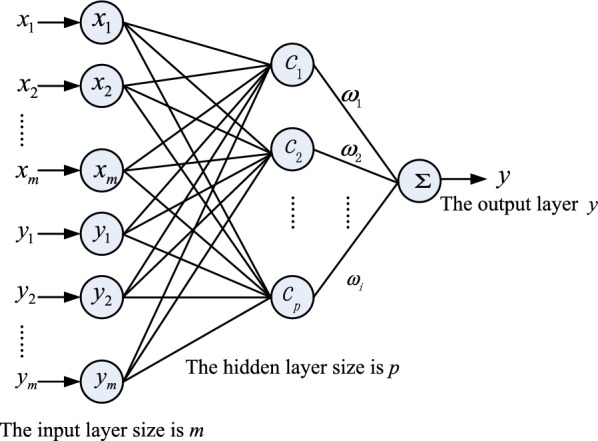
A structure of an RBF network

**Fig. 4:**
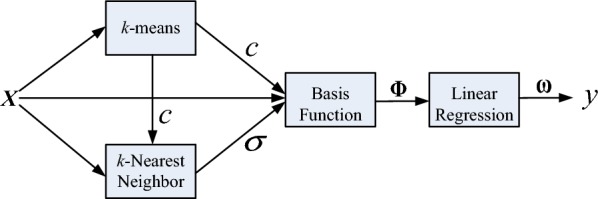
A schematic drawing of the RBF learning process

Any variate *y* can by predicted by a linear combination of a series of RBFs with respect to its past value vector ***Y*** and other past value vector ***X***:

12 $$y=\sum_{i=1}^{ n}{{\boldsymbol{\upomega}}}_{i} \boldsymbol{\Phi }(\cdot )\quad  i=1, 2, \ldots, n$$

where *n* is the total number of RBFs involved. For fitting a nonlinear dynamical relation between different variates, three parameters need to be solved: the center vector *c*, the width $\sigma$, and the output layer weight ${{\varvec{\upomega}}}_{i}$. A parameter learning is designed to obtain the optimal parameters with a high prediction accuracy. See Fig. 3 for a structure of the RBF where includes an input layer, a hidden layer (nonlinear mapping), and an output layer (linear). The whole process of learning algorithm is summarized as shown in Fig. 4. In Fig. 4, a *k*-means clustering algorithm is used to find *p* center vector *c* [20]. Then, a *k*-Nearest Neighbor (kNN) rule is applied to calculate $\sigma$ [21]. Finally, the weight ${{\varvec{\upomega}}}_{i}$ is obtained by a Minimum Square Error (MSE) method [22].

### Two-variate nonlinear Granger causality

Similar to the idea of linear Granger causality, a nonlinear Granger causality based on RBFs can also be defined in the framework of VAR model. The dynamic dependence between time series *x* and *y* is expressed as the following nonlinear autoregressive model:

13 $${x-{\mathbf{V}}}_{11}\cdot \boldsymbol{\Phi }\left({\varvec{X}}\right)+{{\mathbf{V}}}_{12}\cdot\boldsymbol{\Psi }\left({\varvec{Y}}\right)={\varepsilon }_{1}, \quad {\text{var}}\left({\varepsilon }_{1}\right)={\varSigma }_{1}$$

14 $$y{-{\mathbf{V}}}_{21}\cdot \boldsymbol{\Phi }({\varvec{X}})+{{\mathbf{V}}}_{22}\cdot \boldsymbol{\Psi }({\varvec{Y}})={\eta }_{1}, \quad {\text{var}}({\eta }_{1})={H}_{1}$$

where {**V**} are fitting parameters for RBF $\boldsymbol{\Phi }\left({\varvec{X}}\right)\, {\text{or}}\, \boldsymbol{\Psi }\left({\varvec{Y}}\right)$, estimated by the MSE criterion. For example, function vector $\boldsymbol{\Phi }=({\varphi }_{1},\dots ,{\varphi }_{{p}_{1}})$ are ${p}_{1}$ given nonlinear real functions of *m* variates and $\boldsymbol{\Psi }=({\psi }_{1},\dots ,{\psi }_{{p}_{2}})$ are ${{p}}_{2}$ other real functions of *m* variates. The number *p*_*i*_ (*i* = 1, 2) is determined by how many clustering centers are obtained after using the *k*-means method. The notation ${\varepsilon }_{1}$ and ${\eta }_{1}$ denote the prediction error, and the covariance matrix of them is:

15 $$\boldsymbol{\Sigma }=\left(\begin{array}{cc}{\varSigma }_{1}& {\varLambda }_{1}\\ {\varLambda }_{1}& { H}_{1}\end{array}\right)$$

where ${\varSigma }_{1}={\text{var}}\left({\varepsilon }_{1}\right), { H}_{1}={\text{var}}\left({\eta }_{1}\right), {\varLambda }_{1}={\text{cov}}({\varepsilon }_{1},{\eta }_{1})$. As shown in (13), time series ${x}_{k}$ of variate *x* in the present moment *k* can be predicted using the sum of the nonlinear function of time series vector ${{\varvec{X}}}^{ k}$ (before the *k* moment), the nonlinear function of time series ${{\varvec{Y}}}^{ k}$(before the *k* moment) and the forecast error ${\varepsilon }_{1}$.

We proposed a strategy to choose the functions $\boldsymbol{\Phi }$ and $\boldsymbol{\Psi }$, in the framework of RBF methods. For example, functions $\boldsymbol{\Phi }$ are selected in the following three forms:

16 $${\varphi }_{\rho }\left({\varvec{X}}\right)={\text{exp}}(-{\Vert {\varvec{X}}-{\stackrel{\sim }{{\varvec{X}}}}^{\rho }\Vert }^{2}/{2\sigma }_{x}^{2}), \quad \rho =1,\ldots ,{p}_{1}$$

17 $${\varphi }_{\rho }\left({\varvec{X}}\right)=1/(1+{\text{exp}}({\Vert {\varvec{X}}-{\stackrel{\sim }{{\varvec{X}}}}^{\rho }\Vert }^{2}/{\sigma }_{x}^{2})), \quad \rho =1,\ldots ,{ p}_{1}$$

18 $${\varphi }_{\rho }\left({\varvec{X}}\right)=1/\sqrt{{\Vert {\varvec{X}}-{\stackrel{\sim }{{\varvec{X}}}}^{\rho }\Vert }^{2}+{2\sigma }_{x}^{2}}, \quad \rho =1,\ldots,{ p}_{1}$$

where ${\{{\stackrel{\sim }{{\varvec{X}}}}^{\rho }\}}_{\rho =1}^{{p}_{1}}$ are the centers of the data ***X*** clustered by the *k*-means algorithm. The notation ${\sigma }_{x}$ denotes the width of the RBF, which controls the radial range of the function. It is calculated using KNN rule. When the effects of other variate *y* (or *x*) are eliminated both in (13) and (14), the aforementioned nonlinear mutual regression model can be deduced to the form:

19 $$x-{{\mathbf{W}}}_{1}\boldsymbol{\Phi }\left({\varvec{X}}\right)={\varepsilon }_{2}, \quad {\text{var}}\left({\varepsilon }_{2}\right)={\varSigma }_{2}$$

20 $$y-{{\mathbf{W}}}_{2}\boldsymbol{\Psi }\left({\varvec{Y}}\right)={\eta }_{2}, \quad {\text{var}}\left({\eta }_{2}\right)={H}_{2}$$

where ${\varepsilon }_{2}, {\eta }_{2}$ denote the estimated errors, ${{\mathbf{W}}}_{1},\,{{\mathbf{W}}}_{2}$ are the parameter vectors of the fitting model. If the prediction variance ${\varSigma }_{1}<{\varSigma }_{2}$, the prediction of *x* is improved after adding the nonlinear effects of *y*. Then it is believed that *y* has a nonlinear Granger causality on *x*, and the nonlinear causal measurement of *y* on *x* can be expressed as:

21 $${\text{F}}_{y\to x}={\text{ln}}\frac{{\varSigma }_{2}}{{\varSigma }_{1}}$$

Similar to the case of linear Granger causality, if *y* has a nonlinear causal effect on $x$, then ${\varSigma }_{2}>{\varSigma }_{1}, { \text{F}}_{y\to x}>0$.

### Conditional nonlinear Granger causality

**Fig. 5:**
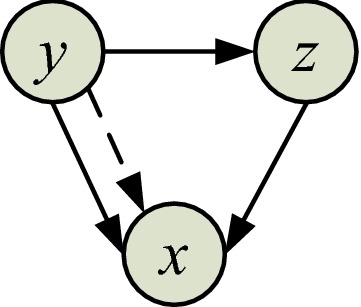
Schematic drawing of conditional causality. For the Granger causalities analysis from *y* to *x*, there is a direct causality and an indirect causality via *z*. All direct connections are denoted by solid lines, and indirect connections are represented by dash lines

In the cases of biological network analysis, the problem usually becomes how to infer functional connections among multivariate network data. At that time, it is unreasonable to only focus on the causal effects between two variates and ignore the effects from other network nodes, such as genes, proteins, metabolites, and neurons. In one BNN, there is often many indirect causalities between two network nodes. Therefore, a test for whether there is a direct drive-response relationship between the two network variates, needs the information from other variates as a condition, known as the "conditional causality" [23] (see Fig. 5 for an illustration).

As shown in Fig. 5, *y* has a direct effect on *z*, and *z* has a direct effect on *x*. The influence of *y* on *x* includes not only the direct influence from *y* to *x* but also the indirect influence through the third variate *z*. The conditional Granger causality test can distinguish between direct and indirect directional effects. Considering the causal effect of *y* on *x* under the condition of the indirect variate *z*, this nonlinear model can be described as:

22 $${x-{\varvec{V}}}_{11}\cdot\boldsymbol{\Phi }\left({\varvec{X}}\right)-{{\mathbf{V}}}_{12}\cdot\boldsymbol{\Psi }\left({\varvec{Y}}\right)-{{\mathbf{V}}}_{13}\cdot \boldsymbol{\Pi }\left({\varvec{Z}}\right)={\varepsilon }_{1},\quad \boldsymbol{ }{\text{var}}\left({\varepsilon }_{1}\right)={\varSigma }_{1}$$

23 $${y-{\varvec{V}}}_{21} \cdot \boldsymbol{\Phi }\left({\varvec{X}}\right)-{{\mathbf{V}}}_{22}\cdot\boldsymbol{\Psi }\left({\varvec{Y}}\right)-{{\mathbf{V}}}_{23}\cdot\boldsymbol{\Pi }\left({\varvec{Z}}\right)={\eta }_{1},\quad {\text{var}}\left({\eta }_{1}\right)={ H}_{1}$$

24 $${z-{\varvec{V}}}_{31}\cdot \boldsymbol{\Phi }\left({\varvec{X}}\right)-{{\mathbf{V}}}_{32}\cdot\boldsymbol{\Psi }\left({\varvec{Y}}\right)-{{\mathbf{V}}}_{33}\cdot \boldsymbol{\Pi }\left({\varvec{Z}}\right)={\upsilon }_{1}, \quad {\text{var}}\left({\upsilon }_{1}\right)={\varUpsilon}_{1}$$

where ${\varepsilon }_{1}\,{\eta }_{1}$, and ${\upsilon }_{1}$ represent the prediction errors, and $\boldsymbol{\Pi }=({\pi }_{1},\dots ,{\pi }_{{p}_{3}})$ are ${p}_{3}$ given nonlinear RBFs of *m* variates. The kernel function can also take the forms of (16–18). To test the direct nonlinear Granger causality from *y* to *x*, there is a need to eliminate the effects of *y* and remodeling the network only using *z* and *x*.

25 $${x-{\mathbf{W}}}_{11}\cdot \boldsymbol{\Phi }\left({\varvec{X}}\right)-{{\mathbf{W}}}_{13}\cdot\boldsymbol{\Pi }\left({\varvec{Z}}\right)={\varepsilon }_{2}, \quad {\text{var}}\left({\varepsilon }_{2}\right)={\varSigma }_{2}$$

26 $${z-{\mathbf{W}}}_{31}\cdot \boldsymbol{\Phi }\left({\varvec{X}}\right)-{{\mathbf{W}}}_{33}\cdot\boldsymbol{\Pi }\left({\varvec{Z}}\right)={\upsilon }_{2}, \quad {\text{var}}\left({\upsilon }_{2}\right)={\varUpsilon}_{2}$$

Under the condition of variate *z*, the measurement of the nonlinear causal effect of *y* on *x* is:

27 $${\text{F}}_{y\to x/z}=\frac{{\varSigma }_{2}}{{\varSigma }_{1}}$$

If there is no direct interaction from *y* to *x* on the condition of *z*, ${{\mathbf{V}}}_{12}$ is a 0 vector, ${\varSigma }_{1}={\varSigma }_{2},{\text{and F}}_{y\to x/z}=$0. Otherwise, *y* has a conditional nonlinear Granger causality on *x* based on the knowledge of *z*, i.e., ${\varSigma }_{2}>{\varSigma }_{1} {\text{and F}}_{y\to x/z}>$0. In this way, when making direct causal judgment between variates through conditional causality tests, the possibility of indirect causal influences should be excluded to ensure the reliability of direct causality tests. It is worthwhile noted that in the process of causal test of conditions with more than 3 variates, variates *z* often need to be extended to all sets of variates except for the current studied variates *y* and *x*.

## Supplementary information

**Additional file 1.** The matlab subroutine to simulate the SNN model in Fig. 1.

**Additional file 2.** The main matlab program for generate the data in Fig. 1.

## Data Availability

The authors have declared that the de-identified datasets used and/or analysed during the current study are available from the corresponding author on reasonable request. Or readers can run the simulation protocol of Pulsed Neural Networks, which are described in our submitted manuscript, to obtain the same data in the manuscript.

## Abbreviations

BNN: Biological Neural Networks
RBF: Radial Basis Functions
NGCIM: Nonlinear Granger Causality Identification Method
SNN: Spiking Neural Networks
IF: Integrate-and-Fire
EEG: Electroencephalography
MEG: Magnetoencephalograph
fNIRS: functional Near-infrared Spectroscopy
fMRI: functional Magnetic Resonance Imaging
IEI: Invasive Electrode Implantation
LGCIM: Linear Granger Causality Identification Method
MEA: Multi-Electrode Array
MI: Mutual Information
DTF: Direction Transfer Function
DBN: Dynamic Bayesian Network
EMA: Evolutionary Mapping Approach
AR: Auto-Regression
VAR: Vector Auto-Regressive
GF: Gauss Function
RSF: Reflected Sigmoidal Function
IMQF: Inverse Multi-Quadrics Function
kNN: *k*-Nearest Neighbor
MSE: Minimum Square Error
